# Oral Fish Collagen Peptide Complex Enhances Skin Rejuvenation and Systemic Health Biomarkers: A 90 Day Prospective Observational Study

**DOI:** 10.1111/jocd.70841

**Published:** 2026-04-09

**Authors:** Jingjing Huang, Chao Chen, Meng Zhang, Jinyong Li, Rongchang Wang, Yuying Wang, Jiachan Zhang, Qing Chen

**Affiliations:** ^1^ School of Light Industry Science and Engineering Beijing Technology and Business University Beijing China; ^2^ Beijing Key Laboratory of Plant Resource Research and Development Beijing Technology and Business University Beijing China; ^3^ Tianmeijian Biotechnology Co., Ltd Native Nutrition and Medical Research Institute Beijing China; ^4^ Jiangsu Tianmeijian Nature Bioengineering Co., Ltd Research and Development Center Nanjing China

**Keywords:** facial aging, fish collagen peptides, nutritional combination, skin

## Abstract

**Objective:**

This study aims to evaluate the effects of Fish Collagen Peptide Nutritional Combination (FCPNC) on facial skin rejuvenation and biochemical indicators, including body mass index (BMI), blood lipids, superoxide dismutase (SOD), and bone mineral density in participants.

**Methods:**

This study involved 29 female participants aged 20–55 years, who consumed FCPNC daily for 90 days. Facial skin changes, including wrinkle percentage, pigmentation area, skin hydration, and elasticity, were assessed before and after the intervention. Additionally, BMI, blood tests, and bone mineral density were measured on Day 0 and Day 90. Participants completed questionnaires on skin, nails, hair, and product satisfaction at Days 30, 60, and 90.

**Results:**

After 30, 60, and 90 days, participants exhibited significant improvements in skin hydration, elasticity, and a reduction in wrinkle area percentage. This nutritional combination not only improved skin health but also yielded systemic benefits, including normalizing BMI in 58.33% of overweight participants. SOD activity increased by 31.03%, reflecting enhanced antioxidant capacity. Additionally, calcium levels rose by 48.28%, suggesting potential bone health benefits. Satisfaction surveys revealed increasing positive feedback, with 93% of participants rating the product favorably by the study's conclusion (score ≥ 4).

**Conclusions:**

These findings suggest that FCPNC may positively influence skin health and overall health status, emphasizing its potential as a functional food. However, further large‐scale randomized controlled trials are necessary to validate these results.

## 1 Introduction

As the largest organ of the human body, the skin serves as the primary barrier against environmental stressors. Skin aging, driven by both intrinsic and extrinsic factors, manifests clinically as dryness, loss of elasticity, deepening wrinkles, and hyperpigmentation [1], with collagen degradation representing one of the most prominent alterations. Collagen, constituting approximately 30% of total body protein, forms a triple‐helical structure that provides structural integrity to connective tissues [2]. Within the skin, Type I and III collagen account for approximately 95% of dermal composition, contributing critically to elasticity and tensile strength [2, 3, 4]. However, the aging process progressively diminishes both collagen quantity and quality, resulting in dermal atrophy and subsequent wrinkle formation [5].

With growing public awareness of skin health and quality of life, dietary interventions to mitigate skin aging have attracted considerable attention [6]. Accumulating evidence has demonstrated the beneficial effects of oral collagen supplementation in promoting skin rejuvenation [7]. Fish collagen peptides (FCPs), derived from marine by‐products through enzymatic hydrolysis, exhibit low molecular weight and high bioavailability, enabling efficient intestinal absorption and targeted dermal accumulation [8, 9]. Upon oral administration, FCPs enhance skin hydration by stimulating hyaluronic acid synthesis and aquaporin expression [10, 11]. Furthermore, preclinical studies have demonstrated their capacity to suppress matrix metalloproteinase expression and increase collagen density [12, 13, 14], while clinical investigations have validated significant improvements in wrinkle reduction, skin structure, and elasticity following prolonged supplementation [15].

Beyond collagen peptides, several other nutritional components have demonstrated promise in maintaining skin health. Coenzyme Q10 (CoQ10), an endogenous lipophilic compound essential for mitochondrial energy metabolism [16], possesses potent antioxidant properties that naturally decline with age [17, 18, 19]. Controlled clinical trials have revealed that daily CoQ10 supplementation reduces wrinkle depth while improving skin smoothness and firmness [20]. Similarly, bovine colostrum lyophilized powder, characterized by its abundance of immunoglobulins [21] and bioactive compounds, exhibits robust antioxidant, anti‐inflammatory, and antimicrobial activities [22]. Emerging research has explored the synergistic effects of combined nutritional interventions, demonstrating that FCPs co‐administered with other bioactive compounds—including ornithine [23], vitamins [24, 25], or CoQ10 [26]—amplify anti‐aging effects on multiple skin parameters such as elasticity, hydration, and dermal density.

Despite these advances, several critical knowledge gaps persist. While individual nutrients and selected binary combinations have been investigated, a comprehensive formulation incorporating multiple synergistic components remains unexplored. Moreover, calcium—a mineral essential for maintaining the epidermal calcium gradient and facilitating keratinocyte differentiation—is frequently deficient in contemporary diets [27], yet its incorporation into anti‐aging formulations has received limited attention. Given that calcium deficiency accelerates skin aging by compromising barrier function [28], there is a pressing need for integrated supplementation strategies that address this overlooked component.

Therefore, the present study aims to investigate the combined effects of a multi‐component nutritional formulation comprising fish collagen peptides, bovine colostrum lyophilized powder, CoQ10, and ultra‐fine calcium on facial skin rejuvenation. Through a 90 day intervention trial, we evaluated changes in key skin parameters, including hydration, elasticity, and wrinkle severity, while simultaneously assessing overall health status through comprehensive physical examinations and validated questionnaires. This research seeks to provide robust evidence for synergistic nutritional strategies in combating skin aging and promoting optimal dermal health.

## 2 Materials and Methods

### 2.1 Study Subjects

**FIGURE 1:**
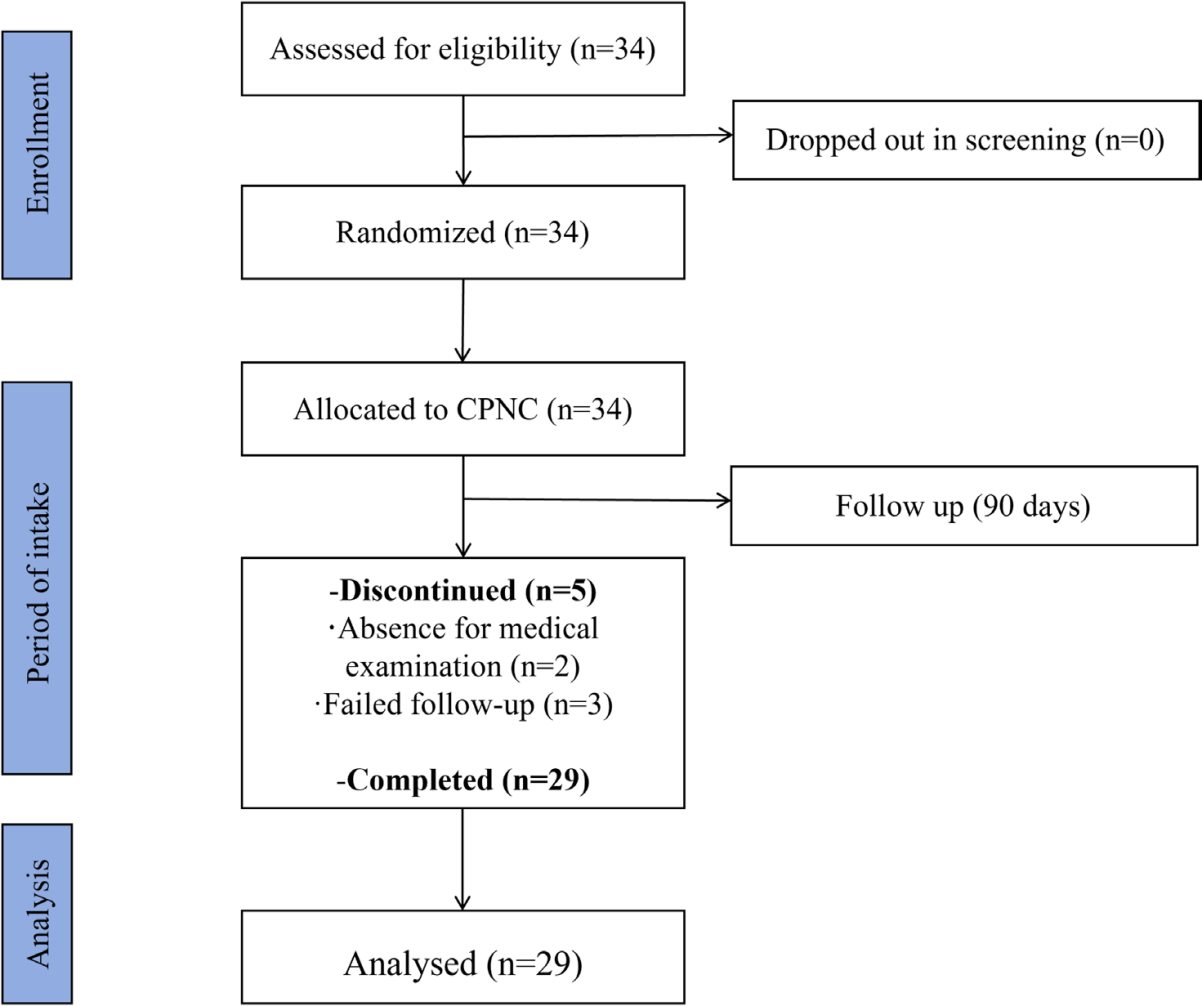
Flow diagram of subjects' selection for the study. Abbreviations: FCPNC, Nutritional combination containing fish collagen peptides.

This clinical investigation was conducted in strict compliance with the Declaration of Helsinki guidelines for biomedical research involving human subjects. A total of 34 female volunteers meeting predefined inclusion/exclusion criteria were initially enrolled through rigorous screening processes. Five subjects withdrew due to personal reasons or protocol deviations, resulting in 29 subjects completing the full study protocol (mean age 34.97 ± 6.69 years; range 25–55 years, Figure 1).

Subjects were required to maintain their habitual facial skincare regimen and cosmetic application practices throughout the observation period, with physical activity levels remaining consistent during the trial.

Inclusion criteria comprised:

1. Healthy female volunteers aged 20–55 years.
2. Presence of clinically observable crow's feet wrinkles and facial hyperpigmentation.
3. Full comprehension of study objectives and procedures.
4. Provision of written informed consent prior to enrollment.

Exclusion criteria were as follows:

1. History of severe systemic diseases.
2. Hypersensitivity to cosmetic ingredients, pharmaceuticals, or food components analogous to test formulation constituents.
3. Recent (≤ 3 months) laser therapy or chemical peeling treatments.
4. Impaired hepatic/renal function or compromised cardiopulmonary capacity.
5. Participation in other clinical trials within 90 days preceding enrollment.
6. Systemic retinoid/corticosteroid therapy within 6 months prior to screening.
7. Topical retinoid application or anti‐aging/keratolytic cosmetic use within 90 days.
8. Current pregnancy or lactation status.
9. Investigator‐determined ineligibility based on clinical judgment.

### 2.2 Test Materials

The method of intake and dosage of all ingredients is in accordance with the instructions. The details are provided in Table 1.

**TABLE 1:** Composition of the test product and intake method.

Product name	Dosage/Day	Active ingredient content	Administration method
Fish collagen peptides	1 vial	12 000 mg/vial, 20 mL/vial	Oral administration
Colostrum freeze‐dried powder	2 sachets	Immunoglobulin 20 000 mg/100 g	Oral administration
Coenzyme Q10	1 capsule	7.46 g/100 g	Oral administration
Calcium and vitamin D3	1 capsule	Calcium 234 mg/capsule, vitamin D3 2.48 μg/capsule	Oral administration

### 2.3 Experimental Design and Observational Protocol

All subjects underwent four scheduled visits to the research center for comprehensive evaluations: baseline assessment (D0) prior to initiating the nutritional intervention, followed by post‐intervention evaluations at 30 days (D30), 60 days (D60), and 90 days (D90). During these visits, standardized anthropometric parameters and skin health indicators were systematically recorded. The safety assessment will be conducted at baseline (D0) and after intervention (D90), including BMI, blood tests, and bone density measurements. Additionally, a subject satisfaction survey was administered within 2 days following the 90 day follow‐up (D90 + 2) to evaluate subjective outcomes. Prior to each evaluation session, subjects adhered to a standardized facial cleansing protocol using a pH‐balanced cleanser, followed by gentle drying with a lint‐free towel. To minimize environmental variability, subjects acclimatized for 30 min in a climate‐controlled chamber maintained at 40%–60% relative humidity and 20°C–22°C. Identical facial regions were examined across all measurement sessions to ensure anatomical consistency, with high‐resolution imaging and biophysical instruments calibrated according to manufacturer specifications.

### 2.4 Skin Indicator Measurements

The skin hydration measurement was conducted using the Corneometer CM825 device (Corneometer CM825, Courage and Khazaka, Germany) to assess the water content of the stratum corneum on the face and the ventral side of the forearm, 5 cm distal to the elbow joint. The probe operates based on capacitive measurement principles, where the dielectric constant of water is 81, and the dielectric constant of other substances is generally less than 7. Water is the substance with the highest dielectric constant on the skin. Changes in water content led to changes in the skin's capacitance value, which allows for the analysis of surface hydration levels through capacitance measurements [29]. The measured value is a relative value, expressed in CU (Corneometer Units). Percentage changes from baseline (D0) were calculated as: [(Follow‐up value − Baseline value)/Baseline value] × 100.

For skin elasticity, one side of the cheek was randomly selected, and the Cutometer MPA580 device (Cutometer MPA580, Courage and Khazaka, Germany) was used to conduct three measurements, with the average value taken. The parameters were set as follows: Pressure 450 mbar; on‐time 2.0 s; off‐time 2.0 s; repetition 2 times. This device operates based on suction and stretching principles, generating negative pressure on the skin surface to draw the skin into a specific test probe. The depth of skin indentation is measured by a non‐contact optical system within the probe, which includes both a light emitter and a receiver. The ratio of emitted to received light correlates with the depth of skin indentation [30]. Using MPA software, skin elasticity is analyzed, with parameters such as the skin elasticity index R2 (maximum roughness), R3 (average roughness), R4 (smooth roughness), and R5 (arithmetic mean roughness). Percentage changes from baseline (D0) were calculated as: [(Follow‐up value − Baseline value)/Baseline value] × 100.

For facial imaging, the VISIA CR imaging system (Canfield Imaging Systems, Fairfield, NY, USA) was used to capture images of the left, center, and right side of the subjects' faces. The light sources were set to Standard 1, Standard 2, Cross‐polarized light, Blue Ex, and Parallel Polarized Light. The following five light sources were used in this test: Standard Light 1: Regular lighting, used to observe the overall condition of the skin; Standard Light 2: Uniform white light illuminating the entire face, removing highlights and shadows; Cross Polarized Light: Polarized light formed through a filter, used to remove surface reflections and observe sub‐surface morphology; Narrowband Blue/UV Light: Blue/UV light formed through a filter, used to capture skin pigmentation; Parallel Polarized Light: Polarized light formed through a filter, used to observe the surface morphology of the skin through surface reflection. The percentage change compared to the baseline (D0) was calculated using the formula: [(Follow‐up value − Baseline value)/Baseline value] × 100.

### 2.5 Physical Examination

The nutritional components were measured at baseline (D0) and 90 days post‐intake (D90). During this period, the participants' BMI was measured, and blood tests were conducted, including the determination of superoxide dismutase activity, four lipid profile indicators (A), fasting blood glucose, vitamin levels (25‐hydroxyvitamin D), and ultrasound bone density scans.

The criteria for judgment are as follows:

*Normal Range:* The measurement value of the indicator falls within the normal range.

*Abnormal Range:* The measurement value of the indicator exceeds the normal range.

*Normal Range Improvement Criteria:* For beneficial indicators (HDL‐C, SOD, 25(OH)D), higher values within the normal range are preferable. Therefore, if these indicators increase within the normal range, it is considered an improvement within the normal range. For harmful indicators (LDL‐C, TC, TG, FPG), lower values within the normal range are preferable. Therefore, if these indicators decrease within the normal range, it is considered an improvement within the normal range. For bidirectional indicators (BMI, BMD), values closer to the midpoint of the normal range are ideal. Therefore, if these indicators approach the midpoint of the normal range, it is considered an improvement within the normal range.

*Abnormal Range Improvement Criteria:* When an indicator exceeds the normal range, if its value returns to the normal range or shows a trend toward the normal range, it is considered an improvement in the abnormal range.

### 2.6 Satisfaction Survey

Each subject evaluated their skin, nails, hair condition, overall well‐being, and the perceived appreciation of the study product on a scale from 1 to 10. The raw individual responses at all four time points (D0, D30, D60, and D90) are detailed in Table S1 (Sheets D0‐D90).

### 2.7 Statistical Analysis

Statistical analyses were performed using GraphPad Prism (version 10.1.2, GraphPad Software, San Diego, USA) statistical software. For descriptive statistics, Microsoft Excel (version 16.0, Microsoft Corporation, Redmond, USA) was used. All data were presented as mean ± standard deviation (SD). Normality was assessed using the Shapiro–Wilk test. For each subject, the percentage change in skin indicators relative to baseline (D0) was calculated as follows: the change rate on Day 30 = (D30‐D0)/D0 × 100%, the change rate on Day 60 = (D60‐D0)/D0 × 100%, and the change rate on Day 90 = (D90‐D0)/D0 × 100%. To evaluate the time effects between D0, D30, D60, and D90, repeated measures analysis of variance (ANOVA) was performed, with Mauchly's test for sphericity. If the sphericity assumption was violated, the Greenhouse–Geisser correction was applied. Significant time effects were further analyzed using paired *t*‐tests with Bonferroni correction (adjusted α = 0.0167) for pairwise comparisons. For data that were not normally distributed, the Wilcoxon signed‐rank test was used to analyze within‐subject differences at different time points. Statistical significance was defined as *p < 0.05, **p < 0.01, ***p < 0.001, and n.s. (no significance) for *p* > 0.05.

## 3 Results

### 3.1 Human Subjects

A total of 34 subjects were enrolled in the study, of which 5 withdrew due to personal reasons or violations of the study protocol (Figure 1). Ultimately, 29 subjects completed the 90 day experiment and follow‐up. No adverse events related to the intake of the test food were reported by any of the subjects.

### 3.2 Skin Hydration

**FIGURE 2:**
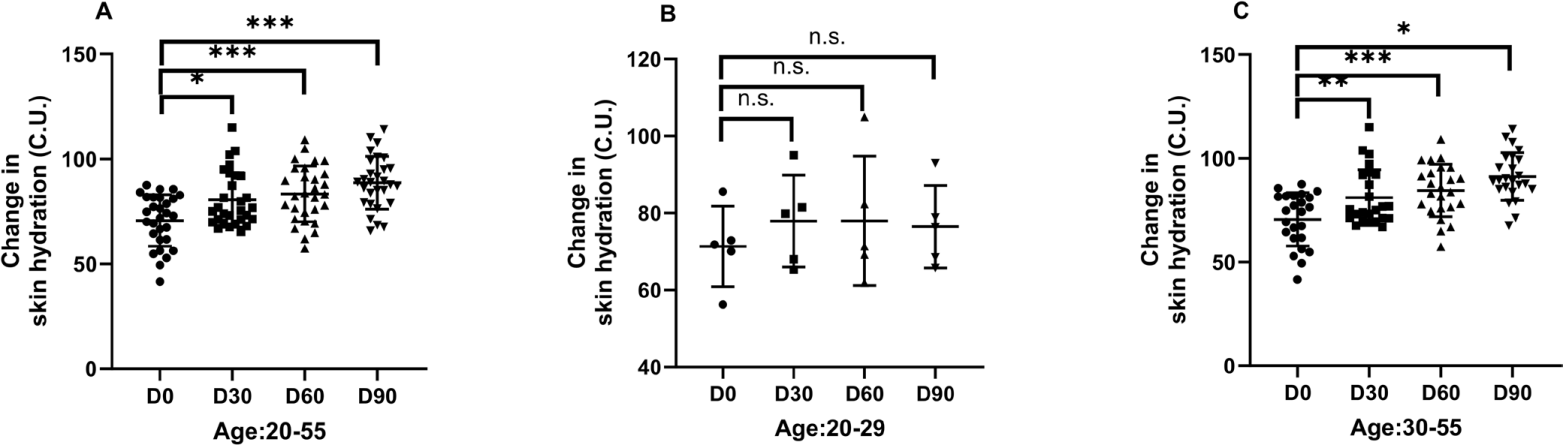
Changes in skin hydration in individuals (A, age range 20–55 years; B, age range 20–29 years; C, age range 30–55 years). Skin hydration was measured using a Corneometer CM825, with changes from baseline expressed in Corneometer Units (CU). Data were presented as means ± SD. *p < 0.05, **p < 0.01, ***p < 0.001, n.s. *p* > 0.05, compared with the baseline (D0).

The longitudinal study demonstrated significant improvements in cutaneous hydration levels among subjects (*N* = 29, age range 20–55 years) at all follow‐up intervals compared to baseline (D0). Remarkably, a 25.41% increase in mean skin moisture content was observed at day 90 relative to baseline measurements (*p* < 0.01, Figure 2A).

Age‐stratified analysis revealed differential response patterns. Subjects in the 30–55 years cohort exhibited statistically significant elevation in skin hydration at all evaluation points (D30: *p* < 0.01; D60: *p* < 0.001; D90: *p* < 0.05, Figure 2C). In contrast, the younger subgroup (20–29 years) showed no significant alterations in cutaneous moisture parameters at any measured timepoint (D30: *p* = 0.38; D60: *p* = 0.47; D90: *p* = 0.84, Figure 2B), suggesting age‐dependent variability in response.

### 3.3 Skin Elasticity

**FIGURE 3:**
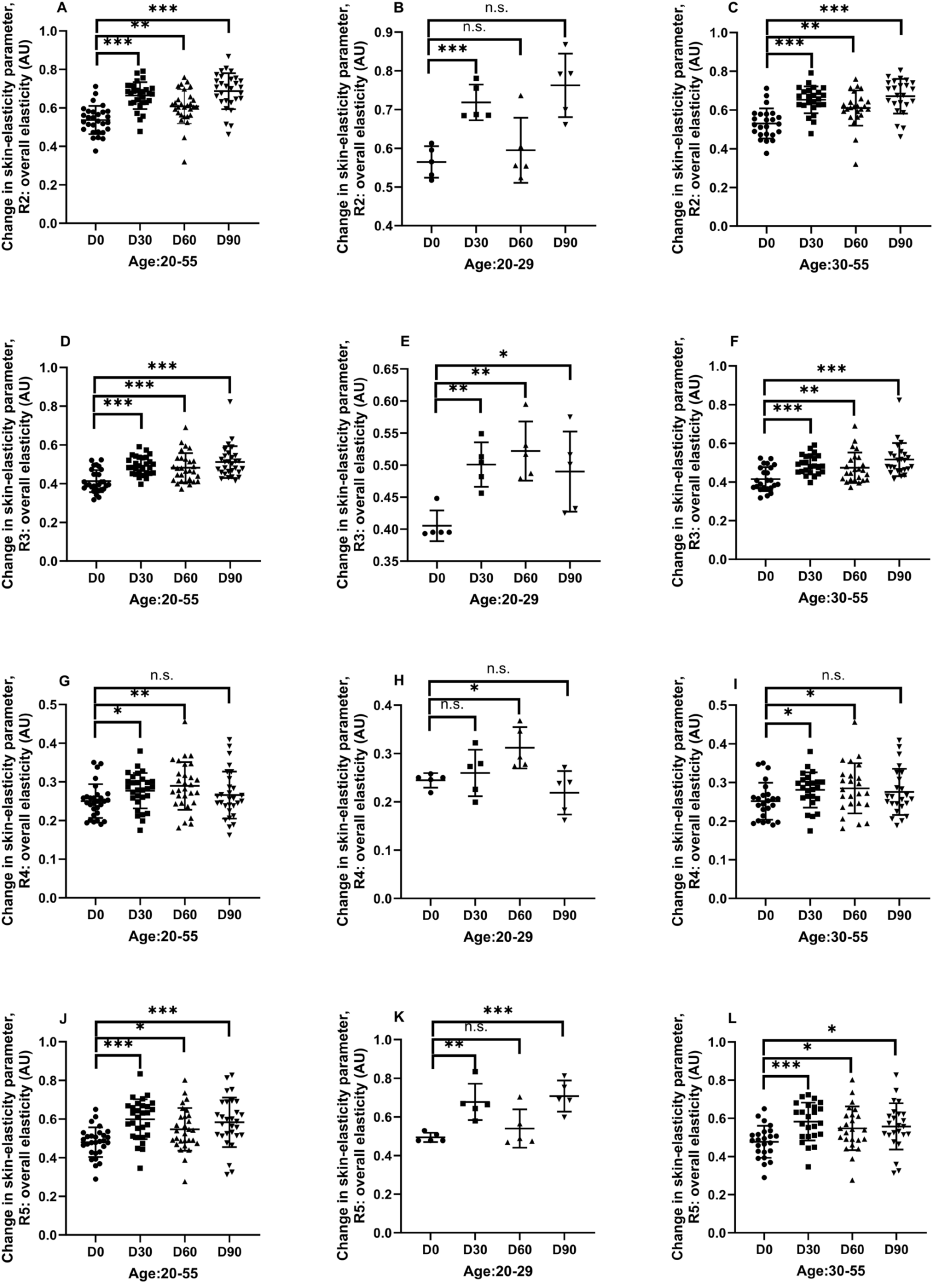
Changes in skin‐elasticity parameters in individuals. (A–C) Changes in skin‐elasticity parameter R2 (maximum roughness). (D–F) Changes in skin‐elasticity parameter R3 (average roughness). (G–I) Changes in skin‐elasticity parameter R4 (smooth roughness). (J–L) Changes in skin‐elasticity parameter R5 (arithmetic mean roughness). R2, R3, R4 and R5 were measured instrumentally with the Cutometer MPA580. Changes in parameter values from baseline are shown in arbitrary units (AU). (A, D, G, and J) Presented the data from the entire study population (20–55 years). (B, E, H, and K) Displayed the data from the younger subgroup (20–29 years); (C, F, I, and L) Showed the data from the older subgroup (30–55 years). Data are presented as means ± SD. *p < 0.05, **p < 0.01, ***p < 0.001, n.s. *p* > 0.05, compared with the baseline (D0).

Skin elasticity measurements were performed on subjects (*N* = 29, age range 20–55 years). Longitudinal analysis revealed a progressive enhancement in skin elasticity R2 values across all follow‐up intervals (D30, D60, D90) relative to baseline (D0), culminating in a 28.02% cumulative improvement (*p* < 0.001, Figure 3A). Age‐stratified analysis demonstrated marked divergence: subjects aged 30–55 years exhibited statistically significant elevations at all timepoints (D30: +22.9%, *p* < 0.001; D60: +15.0%, *p* < 0.01; D90: +26.5%, *p* < 0.001), whereas the 20–29 years cohort showed no significant variation (*p* > 0.05 at all intervals) (Figure 3B,C).

The R3 parameter achieved 23.80% overall augmentation versus baseline (*p* < 0.001, Figure 3D). While the older subgroup maintained consistent upward progression (D30: +18.2%, *p* < 0.001; D60: +14.2%, *p* < 0.01; D90: +24.4%, *p* < 0.001), younger subjects displayed significant increases (D30: +23.6%, *p* < 0.01; D60: +28.8%, *p* < 0.01; D90:‐2.2%, *p* < 0.05) too (Figure 3E,F).

Notably, R4 values exhibited improvement patterns at D30 (+10.7%, *p* < 0.05) and D60 (+15.6%, *p* < 0.01), compared to D0 (Figure 3G). However, at D90, there was no significant difference, though the overall increase was 6.13% (Figure 3H,I).

This contrasted with R5 dynamics, where sustained 21.51% enhancement persisted through final assessment (*p* < 0.001, Figure 3J). Age‐stratified analysis demonstrated slight divergence. The 30–55 years group showed progressive R5 increase (D30: +22.1%, *p* < 0.001; D60: +14.8%, *p* < 0.05; D90: +16.8%, *p* < 0.05, Figure 3L), while younger subjects demonstrated intermittent significance (D30: +37.1%, *p* < 0.01; D90: +4.5%, *p* < 0.001, Figure 3K) with null effect at D60 (+9.2%, *p* = 0.35, Figure 3K).

### 3.4 Skin Pigmentation Analysis and Fine Wrinkles Assessment

**FIGURE 4:**
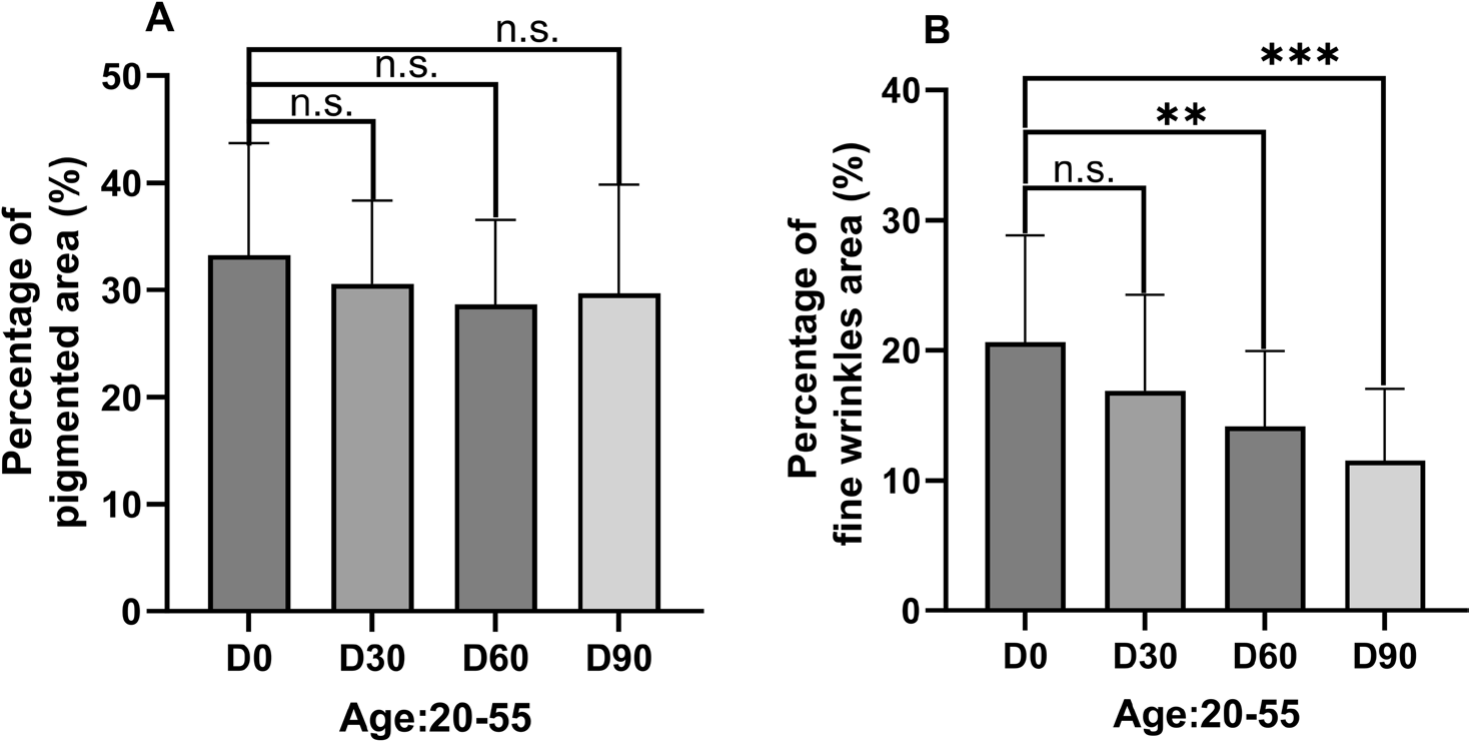
Changes in the percentage of pigmented area (A) and fine wrinkles area (B) in individuals (20–55 years) receiving FCPNC. Quantifications of skin pigmentation and fine wrinkles areas were measured using ImagePro‐Plus image analysis software with standardized illumination protocols. Percentage changes from baseline (D0) were calculated as: [(Follow‐up value − Baseline value)/Baseline value] × 100. Data were presented as means ± SD. *p < 0.05, **p < 0.01, ***p < 0.001, n.s. *p* > 0.05, compared with the baseline (D0).

In the cohort of 29 subjects (age range: 20–55 years), facial skin pigmentation area proportion demonstrated no statistically significant alterations at D30, D60 and D90 follow‐up assessments compared to baseline (D0). However, relative reductions in pigmentation area were observed when calculating percentage changes from baseline means: −8.09% at D30, −13.85% at D60 and −10.83% at D90 (Figure 4A).

Significant improvements in under‐eye fine wrinkles area proportion were recorded at both D60 and D90 time points compared to baseline measurements. The mean percentage changes revealed progressive reductions: −18.11% at D30, −31.42% at D60 (*p* < 0.01), and −44.15% at D90 (*p* < 0.001, Figure 4B). This pattern indicated a time‐dependent improving effect, with maximal efficacy observed at the 90 day evaluation.

**FIGURE 5:**
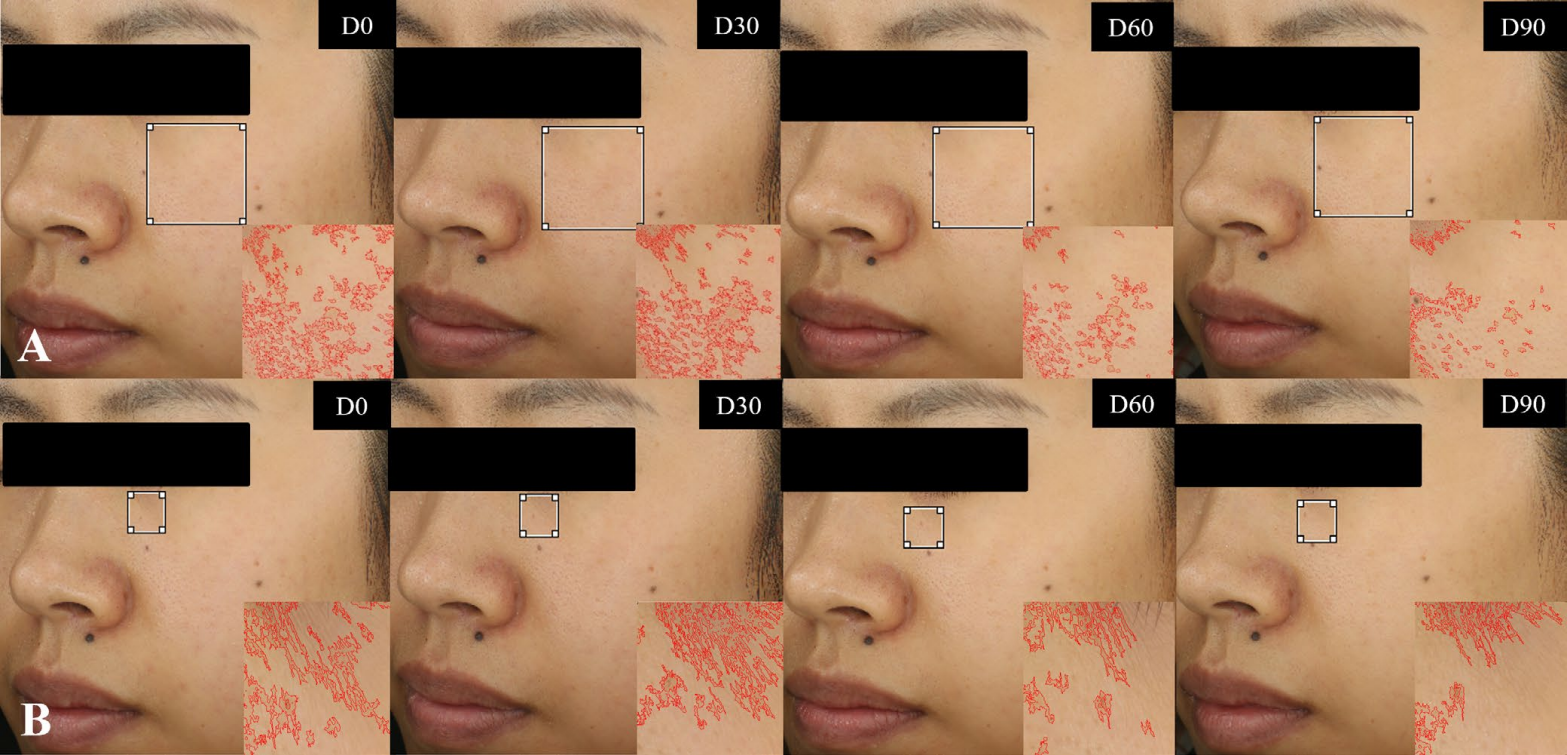
Representative photographs of Subject I showing the visual reduction in pigmentation and fine wrinkles area at D0, D30, D60, D90.

Representative facial photographs documented at D0, D30, D60, and D90 time points following nutritional supplementation demonstrated visible improvements in both pigmentation and fine wrinkles characteristics. A representative case (Subject I) exhibited a 17.54% reduction in facial pigmentation area and a 9.3% decrease in under‐eye fine wrinkles (Figure 5). These clinical findings corroborate the quantitative measurements, suggesting that sustained FCPNC administration promotes progressive dermatological enhancement.

### 3.5 Self‐Evaluation Questionnaire

All 29 subjects completed validated self‐assessment questionnaires evaluating perceived improvements in skin, nails, and hair conditions following test product application, with parallel satisfaction assessments using a 10‐point scale (higher scores denoting stronger agreement/satisfaction). The complete dataset across all evaluation time points is provided in Table S1 (Sheets D0, D30, D60, and D90).

**FIGURE 6:**
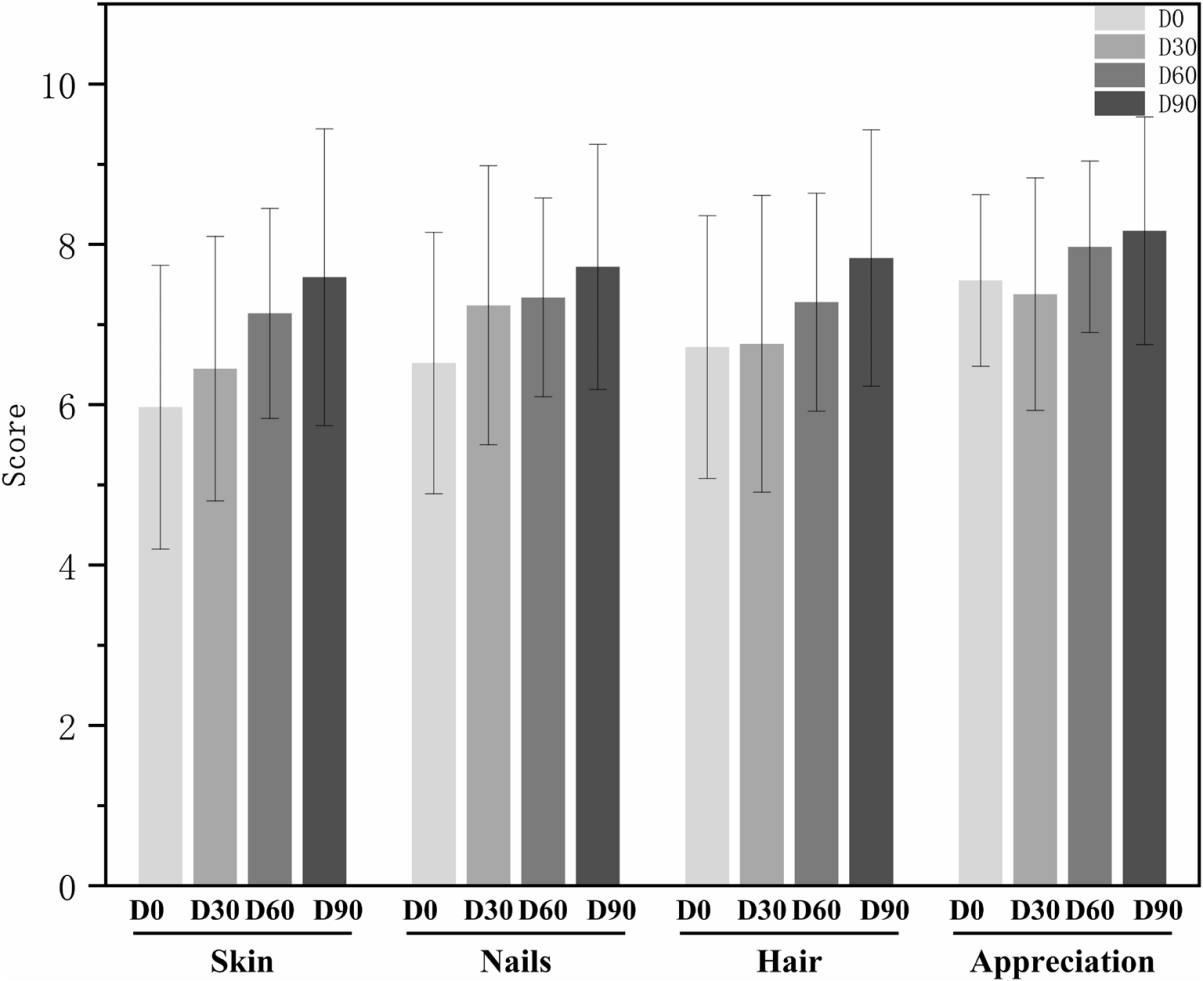
Results of the self‐perception questionnaires completed by all subjects (*n* = 29) regarding their skin, nails, hair, and overall appreciation of the test product after 90 days of treatment. Data were presented as score mean ± SD.

Analysis of the scores revealed a positive correlation between the duration of product use and the reported improvements in skin, nail, and hair condition. The satisfaction results showed that as the duration of product use increased, participants' satisfaction levels rose. The maximum average improvement score for skin increased by 1.3 times from D0 to D90 (Figure 6). Satisfaction indicators also demonstrated a similar trend over time, and by the end of the study, 93% of participants gave positive feedback (score ≥ 4).

### 3.6 Safety Body Checks

Detailed health body checks of 29 subjects were performed before and after a 90 day period to evaluate the potential effects of FCPNC. Body mass index (BMI) was calculated as weight in kilograms divided by height in meters squared. Serum total cholesterol (TC), high‐density lipoprotein cholesterol (HDL‐C), low‐density lipoprotein cholesterol (LDL‐C), and triglycerides (TG) were determined to explore their potential effects on lipid metabolism. Superoxide dismutase (SOD) levels were also evaluated to measure the antioxidant defense. The 25‐Hydroxyvitamin D (25(OH)D) and bone density were determined to evaluate bone health. Meanwhile, fasting plasma glucose (FPG) was measured to evaluate whether the intake of this composition had an impact on the body's blood glucose.

Statistical analysis of the above indicators showed no significant differences between D0 and D90 (Table 2). However, still some subjects showed improvements, typically in BMI, TC, HDL‐C, LDL‐C, 25(OH)D, and BMD after 90 days of supplementation (Table 3). The overall BMI index had little deviation. Although there were still 12 subjects with abnormal BMI, seven of them (58.33%) showed an improving trend. After supplementing the nutritional combination for 90 days, there were 6 cases with abnormal TC in the tested population, which was 5 cases less than that in the original group. The changes in HDL‐C indicators have a similar trend. After 90 days of combined nutritional intake, the number of people with abnormal indicators decreased from 13 initially to 8. Among the population with abnormal HDL‐C at the beginning, 10 subjects showed an improvement after D90. The number of people with normal and abnormal LDL‐C indexes did not change measurably, but the decreasing trend of this index in the two groups of people could still be observed. The antioxidant capacity (SOD) showed improvements within the normal range, with 31.03% (9 out of the total 29 cases) of subjects exhibiting elevated levels, indicating enhanced antioxidant activity. Vitamin D levels also improved overall, with 9 subjects showing an increase, despite a rise in individuals with insufficient levels. Bone health, as assessed through bone density tests, showed an improvement. There was a notable decrease in the number of subjects with low bone mass or osteoporosis, with the number of individuals in the normal range rising to 27, representing a 48.28% overall improvement.

**TABLE 2:** Variation of BMI, and blood test of the subjects.

Indicator	Day	Mean ± SD (*N* = 29)	Significance
BMI (kg/m^2^)	D0 D90	23.25 ± 3.00 23.03 ± 2.84	― 0.771
TC (mmol/L)	D0 D90	4.80 ± 0.86 4.58 ± 0.75	― 0.313
TG (mmol/L)	D0 D90	0.99 ± 0.59 0.99 ± 0.45	― 1.000
HDL‐C (mmol/L)	D0 D90	1.55 ± 0.29 1.45 ± 0.30	― 0.194
LDL‐C (mmol/L)	D0 D90	2.53 ± 0.75 2.44 ± 0.63	― 0.619
SOD (U/mL)	D0 D90	192.13 ± 14.04 188.43 ± 10.70	― 0.263
FPG (mmol/L)	D0 D90	4.93 ± 0.37 4.93 ± 0.31	― 0.936
25(OH)D (mmol/L)	D0 D90	27.56 ± 11.05 27.90 ± 16.06	― 0.926

Abbreviations: 25(OH)D, 25‐hydroxyvitamin D; BMD, bone mineral density; BMI, body mass index; FPG, fasting plasma glucose; HDL‐C, high‐density lipoprotein cholesterol; LDL‐C, low‐density lipoprotein cholesterol; SOD, superoxide dismutase; TC, total cholesterol; TG, triglyceride.

**TABLE 3:** Changes in the number of participants with improvements in body mass index and blood test indicators.

Indicator	Day	Normal range (*n*)	Abnormal range (*n*)	Normal range improvement (*n*)	Abnormal range improvement (*n*)
BMI (kg/m^2^)	D0 D90	18 17	11 12	― 7	― 7
TC (mmol/L)	D0 D90	18 23	11 6	― 11	― 9
TG (mmol/L)	D0 D90	27 27	2 2	― 12	― 2
HDL‐C (mmol/L)	D0 D90	26 21	13 8	― 3	― 10
LDL‐C (mmol/L)	D0 D90	25 26	4 3	― 15	― 3
SOD (U/mL)	D0 D90	27 29	2 0	― 9	― 2
FPG (mmol/L)	D0 D90	29 29	0 0	― 14	― 0
25(OH)D (mmol/L)	D0 D90	22 19	7 10	― 9	― 3
BMD (g/cm^2^)	D0 D90	12 27	17 2	―	― 14

Abbreviations: 25(OH)D, 25‐hydroxyvitamin D; BMD, bone mineral density; BMI, body mass index; FPG, fasting plasma glucose; HDL‐C, high‐density lipoprotein cholesterol; LDL‐C, low‐density lipoprotein cholesterol; SOD, superoxide dismutase; TC, total cholesterol; TG, triglyceride.

## 4 Discussion

The fast‐paced modern lifestyle and increasingly severe environmental pollution have made skin sensitivity and aging more prominent. The growing consumer awareness of the “inside‐out” beauty concept, along with improved scientific understanding of skin health management, highlights the distinctive therapeutic potential of oral beauty products. Unlike topical cosmetics that primarily act on the stratum corneum, oral formulations demonstrate a more profound mechanistic impact through systemic gastrointestinal absorption.

This study demonstrates that oral supplementation with fish collagen peptides combined with colostrum freeze‐dried powder, coenzyme Q10, and ultrafine calcium significantly improves multiple skin health parameters, including hydration, elasticity, and fine wrinkle reduction over 90 days. These findings are consistent with established research on collagen peptide efficacy. Kim et al. [11] and Evans et al. [31] reported similar improvements in skin elasticity and wrinkles following 12 week collagen peptide supplementation, while Proksch et al. [32] demonstrated enhanced dermal matrix synthesis with 2.5 g daily intake for 8 weeks. Particularly, fish collagen peptides, known for their cosmetic efficacy, have demonstrated notable bioavailability advantages [33]. In one study, 71 women taking it for 12 weeks saw significant reductions in periorbital wrinkles and improvements in skin moisture and elasticity [34, 35]. These findings were corroborated by another randomized trial, where a 1000 mg daily dose of low‐molecular‐weight fish collagen peptide led to superior hydration, elasticity, and fewer wrinkles compared to placebo [36]. Campos et al. [15] further validated that fish collagen supplementation effectively enhances skin hydration and structural repair.

While single‐nutrient supplementation remains popular, this study extends prior research by investigating a multi‐component formulation. In contrast to single‐nutrient approaches that typically target specific health aspects, synergistic combinations may promote skin health through multiple pathways. Czajka et al. [24] reported 40% elasticity improvement with collagen peptides combined with vitamins and bioactive compounds over 90 days, demonstrating enhanced collagen fiber structure. Similarly, De et al. [37] found that fish collagen peptides combined with plant‐derived antioxidants (coenzyme Q10, grape skin extract, luteolin, selenium) significantly improved elasticity, sebum secretion, and ultrasound markers. The present study offers a unique contribution by incorporating colostrum freeze‐dried powder and ultrafine calcium, components that distinguish it from existing formulations. This combination showed potential for enhancing antioxidant capacity and appeared to support calcium levels, suggesting that formulation composition dictates functional scope: while collagen‐centric preparations prioritize dermal remodeling, inclusion of specific bioactive compounds and minerals may extend benefits to systemic antioxidative capacity and metabolic homeostasis.

These findings provide novel insights into nutritional interventions for skin rejuvenation beyond dermal effects. The observed BMI reduction in overweight participants and significant cholesterol normalization in hypercholesterolemic subjects suggest broader metabolic benefits. The formulation's capacity to elevate vitamin D and calcium levels while improving antioxidant markers indicates potential for comprehensive health optimization. This multi‐dimensional efficacy may contribute to clinical practice by offering evidence‐based alternatives to single‐target interventions, particularly for populations seeking integrated approaches to skin aging and metabolic health management.

Several limitations warrant consideration. First, the absence of a placebo control group and the inability to strictly regulate lifestyle variables, such as diet and ultraviolet exposure, preclude the definitive exclusion of confounding factors and the establishment of strict causality. Second, the multi‐component nature of the formulation challenges the isolation of specific ingredient contributions versus synergistic effects. Third, the modest sample size and restriction to female participants constrain statistical power and generalizability to broader populations. Finally, the relatively short intervention period and reliance on self‐reported data limit the assessment of long‐term sustainability and introduce potential subjective bias.

Prospective research directions include: first, conducting large‐scale randomized controlled trials to validate efficacy and eliminate confounding variables; second, employing multi‐arm designs and molecular analyses to isolate ingredient contributions and elucidate mechanistic pathways; third, recruiting diverse cohorts, including males and broader age groups, to enhance the generalizability of the findings; and fourth, extending intervention duration to assess temporal stability of observed effects.

Despite these limitations, this study offers substantive evidence supporting oral fish collagen peptide formulations in anti‐aging applications, extending current knowledge by demonstrating synergistic benefits of a novel multi‐component composition on both dermatological and metabolic parameters.

## 5 Conclusions

This study demonstrates that Fish Collagen Peptide Nutritional Composition (FCPNC) significantly improves skin quality and systemic health markers in women aged 20–55 over 90 days. The findings highlight FCPNC's potential as a comprehensive intervention for both dermatological and metabolic wellness.

## Author Contributions

Jia‐chan Zhang and Qing Chen made substantial contributions to the conception and design, acquisition of data, or analysis and interpretation of data. Jing‐Jing Huang and Chao Chen were involved in drafting the manuscript or critically revising it for important intellectual content, and have given final approval of the version to be published. Meng Zhang and Jin‐yong Li participated in the design of the methodology and made significant contributions to data curation and formal analysis. Rong‐chang Wang and Yu‐ying Wang were responsible for funding acquisition and resource allocation, project administration, and contributed to the critical review, commentary, and revision of the manuscript. All authors have participated sufficiently in the work to take public responsibility for appropriate portions of the content. Furthermore, all authors have reviewed and approved the final manuscript and agree to be accountable for all aspects of the work, ensuring that questions regarding the accuracy or integrity of any part are properly investigated and resolved.

## Funding

This work was supported by Tianmeijian Biotechnology Co. Ltd. The funder provided support in the form of salaries for authors C.C., R.W., Y.W., and Q.C., compensation for volunteers, and research materials.

## Ethics Statement

The study was conducted in accordance with the Declaration of Helsinki and approved by the Beijing Technology and Business University Scientific Research Ethics Committee (Approval No. 2024‐169). Informed consent was secured from all subjects prior to their inclusion in the study.

## Consent

All participants provided informed consent, which included the use of personal data, including any personal details or images.

Photo consent: Written informed consent was obtained from the patient/subject for the publication of their photograph.

The manuscript is submitted with the full approval of all listed authors.

## Conflicts of Interest

Qing Chen serves as the Chairman of Tianmeijian Biotechnology Co. Ltd. and Jiangsu Tianmeijian Nature Bioengineering Co. Ltd. Chao Chen, Rong‐chang Wang, and Yu‐ying Wang are employees of these companies. The remaining authors declare no conflicts of interest.

## Supporting information


**Table S1:** Longitudinal self‐assessment data from a 90 day product efficacy study.

## Data Availability

The data that support the findings of this study are available from the corresponding author upon reasonable request.
