# Supplementary material for: Oral Fish Collagen Peptide Complex Enhances Skin Rejuvenation and Systemic Health Biomarkers: A 90 Day Prospective Observational Study
Source: J Cosmet Dermatol. 2026 Apr 9;25(4):e70841. doi: 10.1111/jocd.70841 (PMC13062880; doi:10.1111/jocd.70841)
Supplement: Supplementary file 1 — Table S1: Longitudinal self‐assessment data from a 90 day product efficacy study. [file JOCD-25-e70841-s001.zip › jocd70841-sup-0001-TableS1@Legend for Table S1.docx]

**Legend for Table S1**

**Table S1. Longitudinal self-assessment data from a 90-day product efficacy study.**

This table presents the raw, individual responses from all 29 female participants at four time points: baseline (D0) and after 30 (D30), 60 (D60), and 90 (D90) days of product use. The data was collected via a WeChat-based questionnaire.

**Column Descriptions:**

**A: Number** - Unique identifier for each questionnaire submission.

**B: Submission deadline** - The specific timestamp of submission.

**C: Time required** - The duration (in seconds) taken to complete the questionnaire.

**D: Source** - The platform used for distribution and collection (all entries: WeChat).

**E: Source details** - Further details on the source (all entries: Not Applicable, N/A).

**F: Total score** - The sum of scores from specific satisfaction and improvement questions (columns J-Q for D30-D90; J-M for D0).

**G: 1. What is your real name?** - Participant identifier (initials used for anonymity).

**H: 2. What is your age?** - Participant's age in years.

**I: 3. What's your gender?** - Participant's self-reported gender (all participants: female).

**Self-Assessment Satisfaction Scores (Scored 1-10, where 10 is "Most Satisfied"):**

**J: 5.** Satisfaction with skin condition.

**K: 6.** Satisfaction with nail condition.

**L: 7.** Satisfaction with hair health.

**M: 8.** Overall sense of well-being.

**Post-Usage Product Evaluation (D30, D60, and D90 only; Scored 1-10, where 10 is "Most Satisfied" or "Greatest Improvement"):**

**N: 9.** Overall satisfaction with the product.

**O: 10.** Perceived improvement in skin condition.

**P: 11.** Perceived improvement in nail condition.

**Q: 12.** Perceived improvement in hair condition.

**Notes:**

1.The baseline (D0) data establishes initial states for satisfaction and well-being (questions 5-8).

Follow-up questionnaires (D30, D60, D90) include additional questions on product satisfaction and perceived efficacy (questions 9-12).

2.Participants can be tracked across all time points using their unique initials.

3.The final two rows of each sheet display the Average and Population Standard Deviation (STDEV.P) for the scores in columns J to Q (or J to M for D0), summarizing the central tendency and variability of responses for each question at that time point.
